# Mechanism of action of trabectedin in desmoplastic small round cell tumor cells

**DOI:** 10.1186/s12885-017-3091-1

**Published:** 2017-02-06

**Authors:** S. Uboldi, I. Craparotta, G. Colella, E. Ronchetti, L. Beltrame, S. Vicario, S. Marchini, N. Panini, G. Dagrada, F. Bozzi, S. Pilotti, C. M. Galmarini, M. D’Incalci, R. Gatta

**Affiliations:** 1 Department of Oncology, IRCCS-Istituto di Ricerche Farmacologiche ‘Mario Negri’, Milan, Italy; 2 Experimental Oncology and Pharmacogenomics, IRCCS Fondazione “Salvatore Maugeri”-Istituto di Pavia, Pavia, Italy; 3 Department of Pathology, Fondazione IRCCS Istituto Nazionale Tumori, Milan, Italy; 4 Cell Biology and Pharmacogenomics Department, PharmaMar, Madrid, 28770 Spain

**Keywords:** DSRCT, JN-DSRCT-1, Trabectedin

## Abstract

**Background:**

Desmoplastic small round cell tumor (DSRCT) is a rare and highly aggressive disease, that can be described as a member of the family of small round blue cell tumors. The molecular diagnostic marker is the t(11;22)(p13;q12) translocation, which creates an aberrant transcription factor, EWS-WT1, that underlies the oncogenesis of DSRCT. Current treatments are not very effective so new active drugs are needed. Trabectedin, now used as a single agent for the treatment of soft tissue sarcoma, was reported to be active in some pre-treated DSRCT patients. Using JN-DSRCT-1, a cell line derived from DSRCT expressing the EWS-WT1 fusion protein, we investigated the ability of trabectedin to modify the function of the chimeric protein, as in other sarcomas expressing fusion proteins. After detailed characterization of the EWS-WT1 transcripts structure, we investigated the mode of action of trabectedin, looking at the expression and function of the oncogenic chimera.

**Methods:**

We characterized JN-DSRCT-1 cells using cellular approaches (FISH, Clonogenicity assay) and molecular approaches (Sanger sequencing, ChIP, GEP).

**Results:**

JN-DSRCT-1 cells were sensitive to trabectedin at nanomolar concentrations. The cell line expresses different variants of EWS-WT1, some already identified in patients. EWS-WT1 mRNA expression was affected by trabectedin and chimeric protein binding on its target gene promoters was reduced. Expression profiling indicated that trabectedin affects the expression of genes involved in cell proliferation and apoptosis.

**Conclusions:**

The JN-DSRCT-1 cell line, in vitro, is sensitive to trabectedin: after drug exposure, EWS-WT1 chimera expression decreases as well as binding on its target promoters. Probably the heterogeneity of chimera transcripts is an obstacle to precisely defining the molecular mode of action of drugs, calling for further cellular models of DSRCT, possibly growing in vivo too, to mimic the biological complexity of this disease.

**Electronic supplementary material:**

The online version of this article (doi:10.1186/s12885-017-3091-1) contains supplementary material, which is available to authorized users.

## Background

Desmoplastic small round cell tumor (DSRCT) was first described in 1989 by Gerald and Rosai [1] as a rare and highly aggressive disease that usually occurs in males during adolescence and early adulthood, with an estimated annual incidence rate of 0.1 cases/per 1 million. Only a few hundred cases have been reported worldwide. DSRCT can be described as a member of the family of small round blue cell tumors. All cases of DSRCT harbor the t(11;22)(p13;q12) translocation, leading to fusion of the N-terminal domain of Ewing’s sarcoma gene (*EWS*) to the C-terminal DNA binding domain of Wilms tumor suppressor gene (*WT1*). This unique chromosomal translocation provides the definitive molecular diagnostic marker of DSRCT and creates an aberrant transcription factor, EWS-WT1, which underlies the oncogenesis [2, 3].

The extreme rarity of the disease and the lack of prospective studies mean there is no consensus on the best treatment [4]. The three main treatment modalities are surgical resection (though complete resection is rarely possible), combination chemotherapy (response to conventional doxorubicin-based therapy is very poor), and local radiotherapy. In any case, current treatments for DSRCTs are not curative and do not give long-term survival benefits [2]. Despite aggressive therapy, 3-year overall survival is estimated at 44% and the 5-year survival remains around 15%.

Two case reports, one by A.M. Frezza [4] and another by A.L. Gonzalez [5], support trabectedin, which was active and safe in pre-treated DSRCT patients, all refractory to several lines of chemotherapy, and who remained stable after trabectedin treatment.

**Fig. 1:**
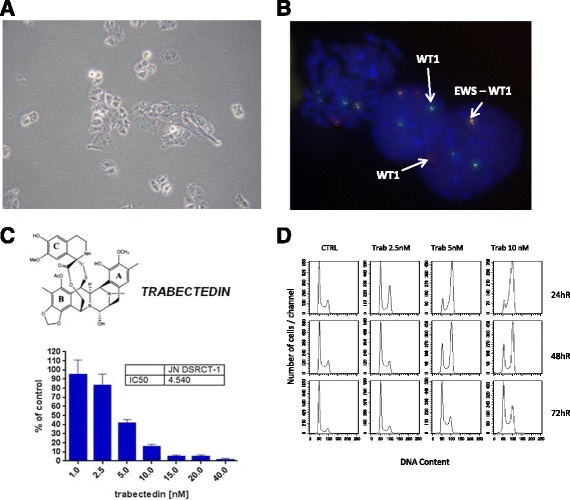
JN-DSRCT-1 cell line resembles DSRCT disease characteristics. **a** 10X image of JN-DSRCT-1 cells. **b** *EWS-WT1* fusion transcripts were detected in JN-DSRCT-1 cells by FISH. Chromosome preparation from JN-DSRCT-1 cells hybridized with WT1 break-apart BAC probes: Spectrum *Orange* labeled RP1- 259 N9 (centromeric WT1 5’ end) and Spectrum *Green* labeled RP11-299P16 (telomeric WT1 3’ end). A fusion signal (corresponding to a non-translocated WT1 allele) with two *green* signals (derivative chromosome 22) and an *orange* signal (derivative chromosome 11) are present in the metaphase andin the interphase nucleus. The FISH pattern is coherent with EWS break-apart (not shown). **c** Trabectedin chemical structure and Clonogenic assay on JN-DSRCT-1 cells. The IC_50_ was calculated by PRISM GraphPad. **d** Cell cycle analysis after 1 h of treatment with trabectedin; the data were analyzed 24, 48 and 72 h after drug wash-out

Trabectedin (ET-743, Yondelis®) is a natural product originally derived from *Ecteinascidia turbinata*, and currently produced semi-synthetically. It has been previously reported that it interacts with the minor groove of DNA through rings A and B, and with several transcription factors, DNA binding proteins and DNA repair molecules presumably through ring C, which protrudes out of the DNA, inducing cell cycle perturbations that finally cause the cancer cell death (Fig. 1c) [6]. It is now used as a single agent for the treatment of soft tissue sarcoma after failure of doxorubicin or ifosfamide, or for patients that are unsuited for these molecules. It is also used in combination with pegylated liposomal doxorubicin for patients with relapsed, platinum-sensitive ovarian cancer. Myxoid liposarcoma, characterized by the expression of the oncogenic transcript FUS-CHOP, was extremely sensitive to trabectedin [6, 7]. Preclinical studies indicated that this sensitivity may be associated with trabectedin’s ability to block the activity of FUS-CHOP chimera, allowing the tumor to differentiate into benign lipoblasts. Displacement of the FUS-CHOP chimera from the promoters of its target genes, blocking its transactivating function, was confirmed in tumor tissues from biopsies of patients with myxoid liposarcomas (MLS) treated with the drug [8]. Thus this event reactivates adipocytic differentiation.

Starting from this assumption, we examined whether DSRCT cells, characterized by the EWS-WT1 chimera expression, are sensitive to trabectedin, as in MLS. Preliminary results already indicate that the drug can be safely used in heavily pretreated DSRCT patients, achieving worthwhile control of symptoms, albeit temporary, with radiological stabilization and regression of disease [4].

JN-DSRCT-1 is an established cell line derived from a primary DSRCT specimen that naturally expresses EWS-WT1 chimera [9]; this human cell line was obtained from the pleural effusion of a 7-year-old boy with pulmonary metastasis from a typical intra-abdominal DSRCT. Cells were small round or spindle-shaped with oval nuclei and have been maintained continuously in vitro for over 190 passages during more than 40 months. Histologic features of the heterotransplanted tumors in the severe combined immunodeficiency mouse were essentially the same as those of the original DSRCT, with nests or clusters of small round cells embedded in an abundant desmoplastic stroma. JN-DSRCT-1 cells exhibited pathognomonic t(11;22)(p13;q12) translocation by cytogenetic analysis. RT-PCR and sequencing analysis showed a chimeric transcriptional message of the Ewing’s sarcoma gene exon 10 fused to the Wilms’ tumor gene exon 8. Alternative splicing in exon 9 of WT1 and EWS-WT1 generates an insertion of three aminoacids -lysine, threonine and serine (KTS)- between zinc fingers 3 and 4, producing + KTS and –KTS isoforms [10]. Both EWS-WT1 -KTS and EWS-WT1 + KTS have been described in DSRCT, though is still not clear from which isoform the oncogenic properties of EWS-WT1 come [11].

Thus, the JN-DSRCT-1 cell line, which presents the morphologic and genetic characteristics of DSRCT, is an in vitro preclinical model useful for studies on the pathogenesis of the disease and for the selection of potential effective drugs.

The aim of our study was the cellular and molecular characterization of one of the in vitro model of DSRCT, JN-DSRCT-1, obtained in S.B. Lee’s laboratory, and investigation of the mode of action of trabectedin in this sarcoma.

## Methods

### Drugs

Trabectedin was provided as a lyophilized formulation by PharmaMar (S.A. Colmenar Viejo, Spain), dissolved in DMSO and stored at -20 °C. Just before use, the drug was diluted in a 1:1 mix of DMEM and Hams F12 medium, supplemented with 10% Fetal Bovine Serum (FBS) and 2 mM glutamine.

### Cell culture

JN-DSRCT-1 cells were grown in a 1:1 mix of DMEM and Hams F12 supplemented with 10% FBS and 2 mM glutamine, in a humidified incubator at 37 °C with 5% CO_2_. This cell line was a kind gift from S.B. Lee.

### RNA extraction, RT-PCR analysis and microarrays

Total RNA was extracted and purified using a commercial kit (miRNAesy Qiagen, Milan, Italy) from 1 × 10^6^ cells; this step was partly mechanized, using an automatic extraction system (Qiacube, Qiagen). The amount of total RNA was determined by UV spectrophotometry using the NanoDrop Spectrophotometer (Nanodrop Technology, Wilmington, USA). One μg of total RNA was reverse-transcribed using the High-Capacity cDNA Archive Kit following the manufacturer’s instructions (Applied Biosystems, California, USA) to assess the differential expression of genes in control cells and cells treated with trabectedin by quantitative real-time PCR (q-PCR). All qPCRs were done in a 25 μl final volume, with three replicates per sample, using QuantiFast SYBR Green PCR kit (Qiagen), and run in an ABI PRISM® 7700 Sequence Detection System (Applied Biosystems). The data were analyzed using the default and variable parameters available in the SDS software package (version 1.9.1; Applied Biosystems). GAPDH housekeeping control gene was used to normalize target gene expression levels and the mRNA amount of each target gene relative to GAPDH was calculated by the comparative Ct method, also called the 2^(−ΔΔCt)^ method. Two biological replicates were each assayed in triplicate and results were expressed as mean ± standard deviation (SD). For microarray experiments, 0.15 μg of RNA were labeled with Cy3 and hybridized according to the manufacturer’s instructions (Agilent Technologies, Palo Alto, CA, USA). After 20 h incubation at 65 °C in rotation at 20 rpm, arrays were washed and scanned with a laser confocal scanner (G2565B, Agilent Technologies, Santa Clara, USA). The microarray underwent standard post-hybridization processing, and signal intensity was calculated with Agilent Feature Extraction (Agilent Technologies), version 11.5 (Agilent Technologies).

Raw data from the scanner were then pre-processed, removing features marked as unreliable by the software, and arrays were normalized using the “quantile” method [12], without background correction. Differentially expressed genes (DEGs) were calculated with linear models applied to microarray analysis [13], correcting the test *p*-value for multiple testing using the False Discover Rate method [14]. Genes were called significant if their corrected *p*-value (*q*-value) was lower than 0.05, corresponding to a 5% maximum of false positives.

Functional enrichment analysis used Fisher’s Exact Test [15] and topological methods [16]. Pathways were called significant if the test corrected *p*-value was 0.05 or lower. Putative network reconstruction based on the scientific literature was done with Ingenuity Pathway Analysis (IPA; Qiagen, USA). Clustering analysis was carried out using the Pearson’s correlation coefficient as a measure of similarity, and with complete linkage.

In accordance to the MIAME guidelines, microarray data were submitted to Array Express (ID E-MTAB-4532).

### Fluorescent in situ hybridization

Fluorescence in situ hybridization (FISH) was used to detect the *EWS-WT1* fusion. FISH was done on chromosome preparations from JN-DSRCT-1 cell line. EWS gene status was assessed by the Vysis EWSR1 dual color break-apart probe (Abbott Molecular, Illinois, USA). WT1 gene status was assessed by the break-apart approach using in-house labeled BAC clones (obtained from C.H.O.R.I. Children’s Hospital Oakland Research Institute): RP1- 259 N9 mapping at the WT1 5’ end and RP11-299P16 mapping at the WT1 3’ end labelled in Spectrum Orange and Spectrum Green (Abbott Molecular), respectively. Probe labeling was carried out according to the manufacturer’s instructions. Slide treatment and FISH experiments were run using standard procedures [17].

### Clonogenic assay, pharmacological treatments and cell cycle analysis

The JN-DSRCT-1 cells were seeded in six-well plates (Eppendorf, Hamburg, Germany) at a concentration of 3500 cells/ml. The clonogenic assay was done as previously described [18]: cells were treated with different concentrations of trabectedin for 1 h and the formation of colonies was evaluated after 5 days. The IC_50_ for each drug was calculated with GraphPad PRISM 6.0. The cell cycle perturbations induced by trabectedin were evaluated by standard flow cytometric methods using FACS Calibur. Control and treated cells were counted using a Coulter Counter (ZM, Beckman Coulter, Brea, CA, USA) after 1 h of treatment and, 24, 48 and 72 hs after drug washout and fixed in 70% ethanol before DNA staining. Data was analyzed using Cell Quest.

### Sanger sequencing method

Total RNA was extracted with an RNeasy Mini Kit (Qiagen) from about 2 × 10^6^ JN-DSRCT-1 cells. Total RNA was determined by UV spectrophotometry using the Nano Drop Spectrophotometer (Nanodrop Technology) and 500 ng of total RNA were reversed-transcribed using the High-Capacity cDNA Archive Kit following the manufacturer’s instructions (Applied Biosystems). Touchdown PCR reactions were run in a final volume of 20 μl of 1× PCR Buffer (5× Colorless Go Taq Flexi Buffer, Promega, Milano, Italy), 200 μM dNTPs mix (Sigma Aldrich, Milano, Italy), 1.5 mM MgCl_2_ (Magnesium Chloride Solution, 25 mM, Promega), 0.5 U GoTaq Hot Start Polymerase (Promega), 50 ng of JN-DSRCT-1 of first strand cDNA, 0.5 μM primer #1 (FW: 5’-TCCTACAGCCAAGCTCCAAGT-3’, Tm = 59.8 °C; Eurofins Genomics Europe, Ebersberg, Germany) and 0.5 μM primer #3 (RV: 5’-ACTTTTTCTGACAACTTGGC-3’, Tm = 53.2 °C; Eurofins Genomics Europe) belonging respectively on the EWS (3’-end of exon 7) and WT1 (5’-end of exon 10) regions of the EWS-WT1 chimera (Additional file 1). Amplification was done in a C1000 Thermal Cycler (Bio-Rad, Segrate, Italy) using the following thermal profile: 2‘at 95 °C for 1 cycle, 30” at 95 °C, 30” at 63 °C (with 0.5 °C decrease at each cycle) and 40” at 72 °C for 26 cycles, 30” at 95 °C, 30” at 50 °C and 40” at 72 °C for 14 cycles, 5’ at 72 °C for 1 cycle; the amplicons of expected size ~320 bp and ~530 bp, corresponding to transcript variants A and B respectively, were separated by 2% 1× TAE buffer agarose gel electrophoresis. To have a pure solution of each transcript variant for the Sanger sequencing, a small amount of each band was picked up from gel with a p200 pipette-tip, eluted for at least 3 h in 20 μl of PCR-grade ddH_2_O and subjected, individually, to Touchdown PCR amplification as described above (using 1 μl of each eluted product as template). The quality and the amount of the pure ~320 bp and ~530 bp amplicons was verified by 2% 1× TAE buffer agarose gel electrophoresis loading a small volume (about 3 μl) of each Touchdown PCR. The remainder of each PCR amplification was purified using the QIAquick PCR purification Kit (Qiagen). Both strands of the two purified variants were Sanger sequenced in a 3130*-16* Genetic Analyzer using the appropriate primer (Additional file 1 Primer #4, FW: 5’-AGAAACCATACCAGTGTGAC-3’, Tm = 55.3 °C; Eurofins Genomics Europe Primer#2, REV: 5’-ACCTTCGGTTCACAGTCCTTG-3’, Tm = 59.8 °C; Eurofins Genomics Europe) and the BigDye Direct Cycle Sequencing Kit, according to the manufacturer’s instructions (Thermo-Fisher Scientific, Monza, Italy).

### Animals and in vivo experiments

To obtain the desmoplastic xenografts, 200 μl of cell suspension containing 5 × 10^7^ cells were injected s.c. into the flanks of NOD- SCID GAMMA (NSG) mice. Desmoplastic xenografts were then obtained by transplanting 2–3 mm tumor fragments s.c. in the flanks of nude mice.

### Chromatin immunoprecipitation analysis (ChIP)

Cells and tumors were crosslinked with 1% formaldehyde. After quenching with glycine, chromatin was sheared by sonication (Bioruptor Diagenode, Seraing, Belgium) in fragments around 500–800 bp. Chromatin immunoprecipitations were done using mouse monoclonal EWS G-5 (sc28327), rabbit polyclonal WT1 C-19 (sc192) and rabbit IgG (7074S, Cell Signaling, Danvers, USA) as negative control. The ChIPped DNA was then re-suspended in water and analyzed by RT-PCR. The detailed procedure of Chromatin Immunoprecipitation analysis was described by Di Giandomenico et al [8]. Data were analyzed according to the fold enrichment method. The sequences of the primers are listed in Additional file 2.

### Western blot analysis

JN-DSRCT-1 cells were treated with 5nM trabectedin for 1 h. After 48 and 72 h drug washout, total extracts were prepared from 2 × 10^6^ cells collected in Ripa Buffer (50 mM TRIS-HCl, pH 7.5, 150 mM NaCl, 1 mM EDTA 500 mM, 1% Triton, 1 mM DTT, 1 mM PMSF, 1× PIC); after centrifuged 10 min at 13,000 rpm 4 °C, the supernatants were stocked at -80 °C. 20 μg of total extracts were used in a SDS-PAGE. Proteinswere transferred to nitrocellulose and analyzed by western blotting with mouse monoclonal EWS G-5 (sc28327), rabbit polyclonal WT1 C-19 (sc192) and goat polyclonal ACTIN (C-11) (sc1615).

### Statistical analysis

We used GraphPad PRISM 6 software for statistical analysis. For RT-Q-PCR data we used the unpaired *t* test and changes were considered significant at a *p* value <0.005. For ChIP RT-Q-PCR we used the paired *t* test and changes were considered significant at a *p* value <0.005.

## Results

### The JN-DSRCT-1 cell line recapitulates DSRCT disease characteristics

We characterized the JN-DSRCT-1 cell line, which derived from a patient with DSRCT [9]. This cell line grows well in vitro with a doubling time of 36 h. Its morphology was confirmed: the shape of the cells was similar to that in biopsies from patients (Fig. 1a). Moreover, JN-DSRCT-1 cells express the EWS-WT1 chimera, as demonstrated by FISH analysis in Fig. 1b, where nuclei in interphase of JN-DSRCT-1 cells clearly reveal the t(11;22)(p13;q12) translocation, leading to the fusion protein EWS-WT1.

### JN-DSRCT-1 cells are sensitive to trabectedin

We further characterized the DSRCT cell line, testing its sensitivity to trabectedin. The clonogenic assay showed that JN-DSRCT-1 cells are very sensitive to trabectedin, with an IC_50_ around 4.5 nM (Fig. 1c). Cell cycle analysis indicated that 2.5 nM (the IC20 at 24 h after drug washout) of trabectedin for 1 h induced a G2/M block already evident at 24 h after drug wash-out. This block was overcome 72 h after drug wash-out. Higher doses of trabectedin, 5 and 10 nM, that represent the IC40 and IC50, respectively, induced cell cycle perturbations that were still partially present 72 h after drug wash-out (Fig. 1d). We do not have any evidences of apoptotic activation, as assessed by biochemical assay (i.e. caspase activity assay, data not shown).

### JN-DSRCT-1 cells present heterogeneity of EWS-WT1 transcripts

**Fig. 2:**
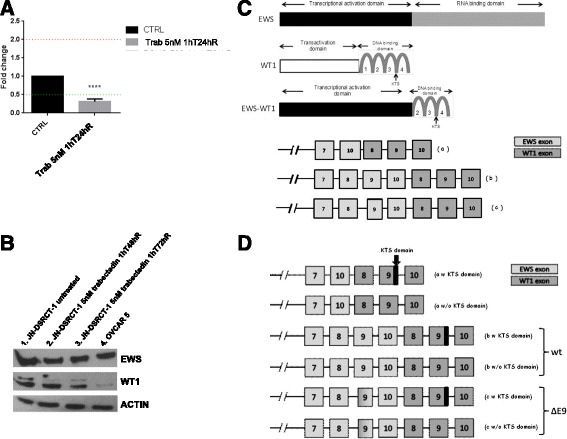
EWS-WT1 transcript heterogeneity in JN-DSRCT-1 cell line. **a** mRNA levels of EWS-WT1 chimera before and after 1 h of trabectedin 5 nM and 24 h after drug wash-out. Data are shown according to the Fold change method using PRISM GraphPad software.* *p* value < 0.005. **b** EWS-WT1 protein levels detected with antibodies against EWS (G-5, sc28327), WT1 (C-19, sc192), ACTIN (C-11, sc-1615). Western blot analysis of total extracts deriving from: lane 1, JN-DSRCT-1 cells untreated; lane 2, JN-DSRCT-1 cells 1hT48hR (1 h 5nM trabectedin treatment and 48 h after drug wash-out); lane 3, JN-DSRCT-1 cells 1hT72hR (1 h 5nM trabectedin treatment and 72 h after drug wash-out); lane 4, OVCAR5 cells. **c** EWS-WT1 chimeric structure diagram and different isoforms of EWS-WT1 chimera, in JN-DSRCT-1 cells. **d** EWS-WT1 variant transcripts with or without KTS domain in JN-DSRCT-1 cells

We checked the mRNA level of EWS-WT1 transcripts by Q-RT-PCR and protein levels by Western Blot. We designed ad hoc primers amplifying EWS-WT1 transcripts, using a forward primer annealing on exon 7 of EWS and a reverse primer annealing on exon 8 of WT1, so we could distinguish the single endogenous proteins from the chimeric ones. We ran RT-Q-PCR on mRNA extracted from JN-DSRCT-1 cells, before trabectedin treatment and after 1 h of 5 nM treatment and 24 h of drug wash out, and verified the expression of EWS-WT1 transcript and protein (Fig. 2a-b): mRNA level of the EWS-WT1 chimera was downregulated, but not the protein levels. Next, to characterize the chimera transcript better, we did a DNA Sanger sequencing. The simultaneous presence of two major transcripts emerged, the first characterized by the fusion of exons 1–7 and 10 of EWS gene to exons 8–10 of WT1, marked in Fig. 2b as (*a*), and the second by the fusion of exons 1–10 of EWS gene to exons 8–10 of WT1 gene, marked in Fig. 2b as (*b*). Analysis of transcript *b* showed that almost 50% of this variant contained a small deletion (3 bp: AGC = > Ser) in the 5’ end of Exon 9 of EWS gene, marked as (*c*). All these variants were present in two sub types, with or without the KTS (Lys-Thr-Ser) domain, located between the zinc finger 3 and 4 of exon 9 of WT1 gene (Fig. 2c). Liu et al. also reported these different isoforms in a study of 14 DSRCT tumors [19]. Thus, the JN-DSRCT-1 cell line showed broad heterogeneity in EWS-WT1 transcripts.

### Trabectedin’s effects on EWS-WT1 expression and function

**Fig. 3:**
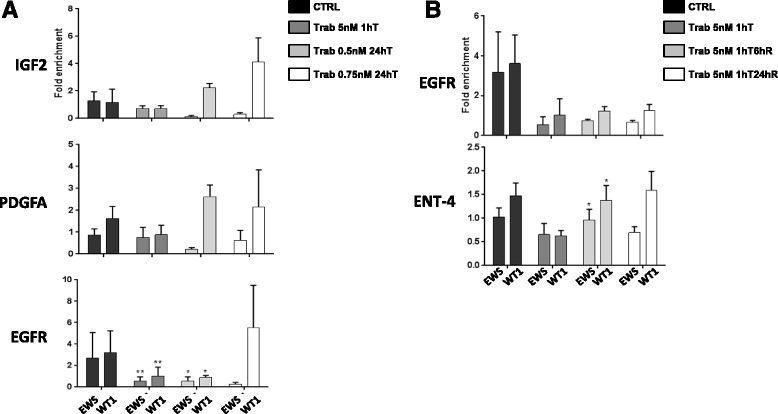
Trabectedin displaces EWS-WT1 chimera from its target gene promoters. **a** ChIP assays on JN-DSRCT-1, treated and not with trabectedin 5 nM for 1 h, 0.5 nM and 0.75 nM for 24 h. The binding of EWS-WT1 on IGF2, PDGFA and EGFR was analyzed by RT-Q-PCR. Data are shown according to the Fold enrichment method using PRISM GraphPad software. * *p* value < 0.005. **b** ChIP assays on JN-DSRCT-1, treated and not with trabectedin 5 nM for 1 h, and recovered after 6 and 24 h. The binding of EWS-WT1 on EGFR and ENT-4 was analyzed by RT-Q-PCR. Data are shown according to the Fold enrichment method using PRISM GraphPad software. * *p* value < 0.005

We examined whether trabectedin reduced the EWS-WT1 chimera binding on its target promoters, through Chromatin Immunoprecipitation (ChIP) assays. We treated cells with 5 nM trabectedin for 1 h, and with 0.5 and 0.75 nM for 24 h. We analyzed three different promoters belonging to EWS-WT1 published target genes, IGF2, PDGFA and EGFR [10]. Trabectedin significantly reduced EWS-WT1 binding, particularly on the EGFR promoter after 1 h with 5 nM trabectedin and 24 h with 0.5 nM trabectedin (Fig. 3a). Then we investigated the kinetics of reattachment of the chimera, on cells recovered 6 and 24 h after treatment: trabectedin kept the reduced binding of the chimeric protein on the EGFR promoter but not on ENT-4 promoter, another target gene bound by EWS-WT1 chimera [3], where the aberrant trascription factor binding was restored already 6 h after drug wash-out (Fig. 3b).

### Expression profiling of JN-DSRCT-1 cells treated with trabectedin suggests an arrest in tumor progression

To determine whether trabectedin altered the expression of EWS-WT1 target genes, we did a whole-transcriptome expression profiling of JN-DSRCT-1, treated with 5 nM trabectedin 6, 12 and 24 h after drug wash-out. One of the control samples failed the quality checks and was discarded.

Unsupervised clustering analysis (Additional file 3) showed an almost unambiguous separation of the control samples from those treated with trabectedin, indicating that the changes in gene expression elicited by the drug were sufficiently higher than the signal background to warrant further analyses.

Differentially expressed genes (DEGs) were then calculated by comparing each time against the untreated control (Additional file 4).

We then analyzed differentially expressed genes (DEGs) 6, 12, and 24 h after drug wash-out. The number of DEGs was higher at 6 h (1417 unique DEGs) when compared to 12 and 24 h (244 and 26, respectively), suggesting the presence of off-target effects at later time points. Thus, to better identify on-target drug effects, we focused on the analysis at 6 h after drug wash out. This analysis identified 1417 unique DEGs (619 up-regulated, 798 down-regulated), including up-regulation of several caspase genes (CASP3, CASP8, CASP10) and down-regulation of PDGFa (Additional file 4). Functional analysis on the DEGs identified the activation of apoptosis-related pathways (p53 signaling and effectors, caspase cascades), activation of cytokine-related pathways (cytokine-cytokine receptor interaction, TNF signaling) and the inhibition of Rap and Ras-related pathways (Additional file 5).

**Fig. 4:**
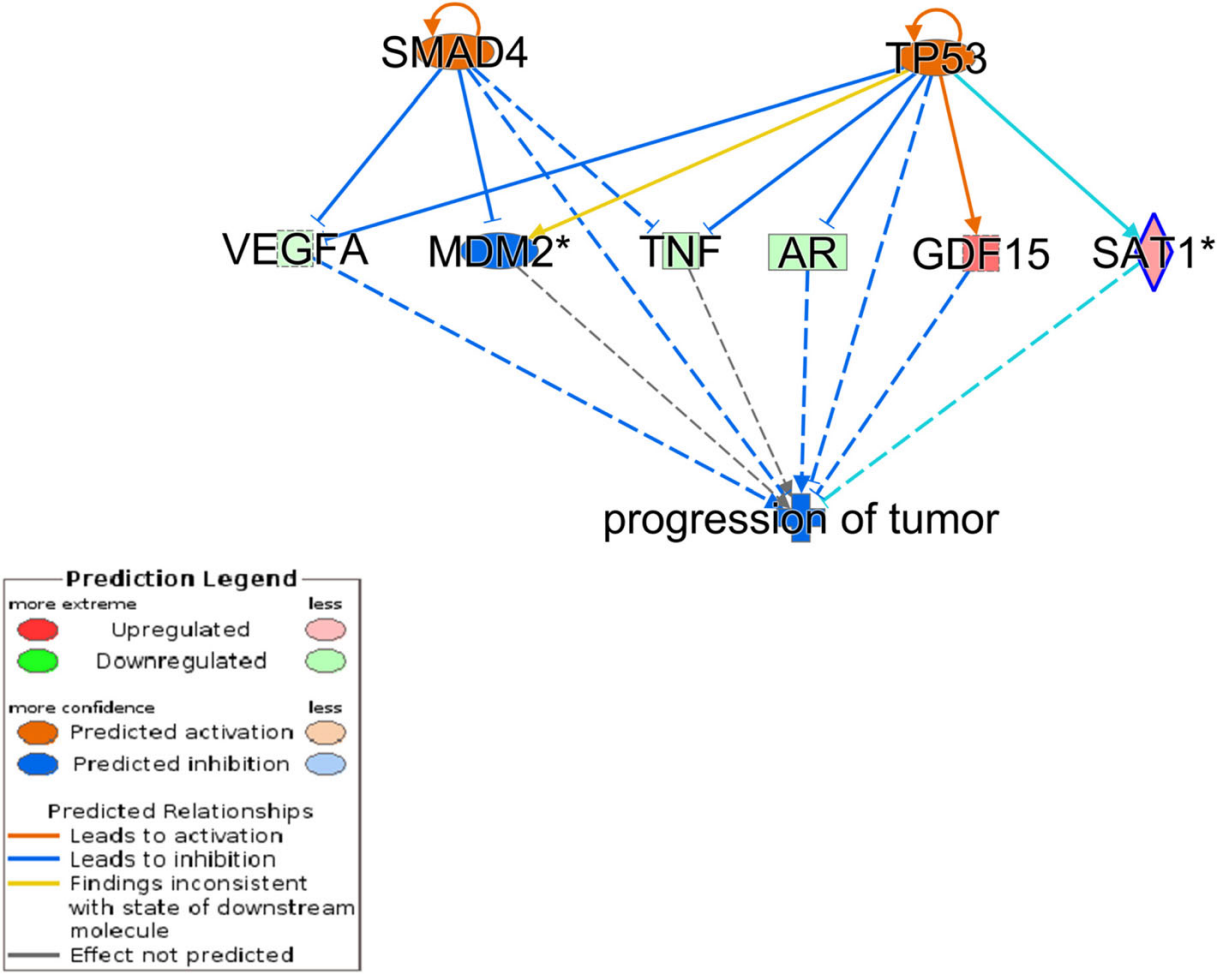
Network analysis of differentially expressed genes (DEGs) after 6 h of trabectedin treatment versus untreated control indicates an inhibition of tumor progression. *Solid arrows* indicate direct interaction, *dashed lines* indirect interaction. *Green*, down-regulated gene in the data set; *red*, up-regulated gene in the data set; *orange*, predicted activation inferred from the state of the present genes; *blue*, predicted inhibition inferred from the state of the present genes. *Blue lines* between genes indicate that the process leads to inhibition, while *orange lines* indicate activation. *Grey lines* indicate absence of any significant predicted effect

To gain additional information on the processes involved, we reconstructed networks based on interactions taken from the current literature using IPA (Fig. 4). Down-regulation of TNF, AR, VEGFA and the up-regulation of GDF15 and SAT1 were part of a predicted network involving SMAD4, MDM2 and TP53, known to be involved in the inhibition of tumor proliferation and progression.

These results suggest that the bulk of the expression changes led by trabectedin on JN-DSRCT1 occur in the early time points, and that the treatment induces mechanisms related to apoptosis (p53 signaling, caspase cascades) and inhibition of proliferation.

### In vivo experiments

**Fig. 5:**
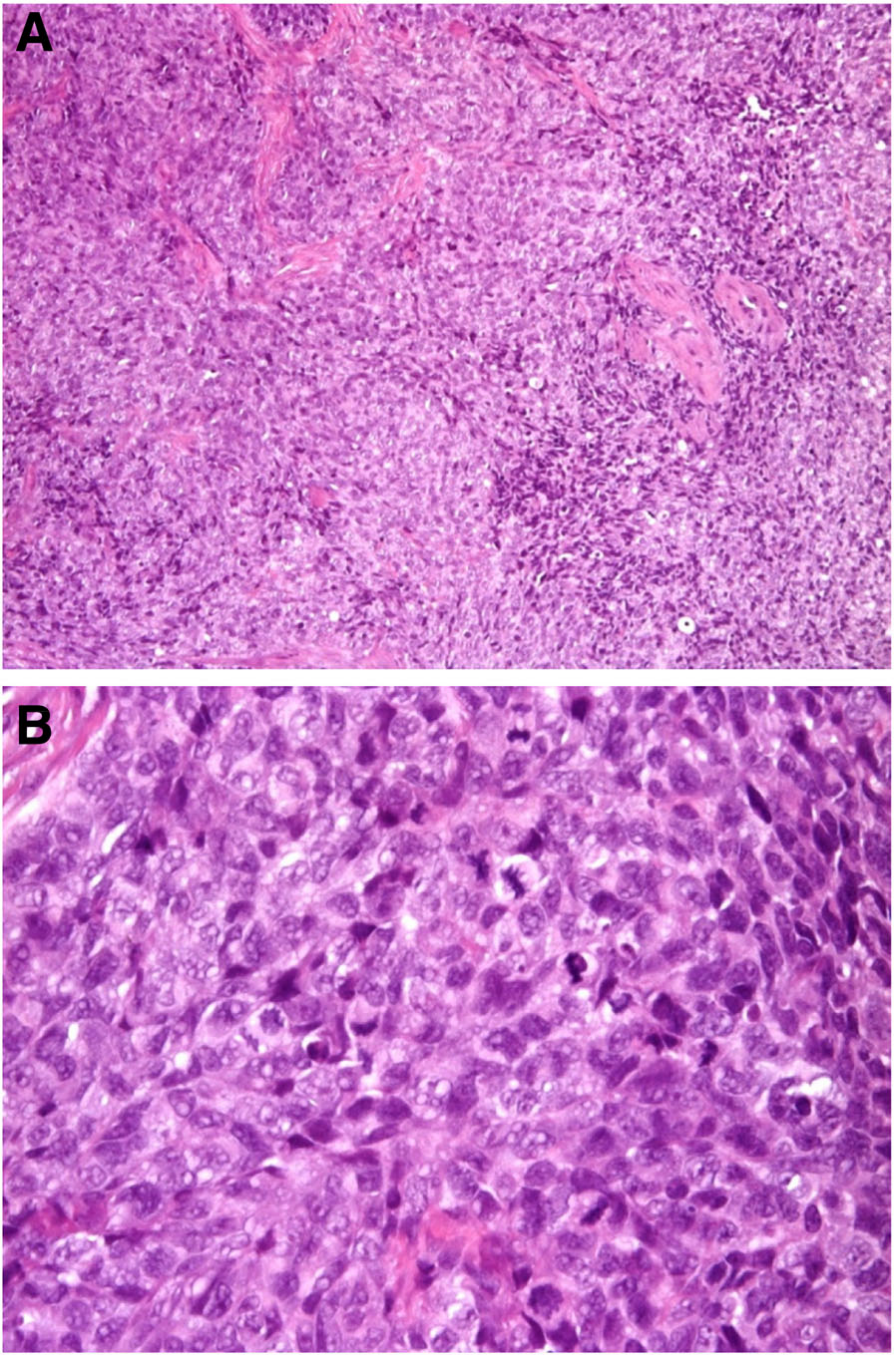
Hematoxylin/eosin staining on the JN-DSRCT xenograft tumors. Biopsies showing a high cellular tumor characterized by a low amount of sclerosis component. **a** 100X section; **b** 200X section

We could not obtain growth of JN-DSRCT-1 inoculated in nude mice, so we transplanted desmoplastic sarcoma cells in more immunodeficient nude SCID mice, observing a tumor take in a small percentage; sections of tumors, from these DSRCT xenograft models, had monomorphic short spindle cells arranged in solid nests with scanty desmoplastic interposed stroma (Fig. 5). The lack of reproducible tumor growth made it difficult to further investigate the activity and the mode of action of trabectedin in these xenografts.

## Discussion

The EWS-WT1 is considered the pathogenic lesion of DSRCT. It is composed of the N-terminal domain of the EWS gene and of the C-terminal DNA binding domain of the WT1 tumor suppressor gene [11]. EWS encodes a putative RNA binding protein of unknown function, whose N-terminal domain mediates potent transcriptional activation when fused to heterologous DNA binding domains. The fusion protein activates a limited number of target genes that are sufficient to trigger malignant proliferation. Since the EWS-WT1 chimera does not contain the first of the four zinc fingers of WT1, it has been reported that an altered DNA binding domain may be essential for cellular transformation in DSRCT. Moreover, the distinct transactivation domain conferred by the N-terminal domain of EWS is likely to lead to interactions with other components of the transcriptional machinery, which may be recruited to target promoters, thus contributing to different targets for these related transcription factors.

We have confirmed that JN-DSRCT-1 cells, which are one of the in vitro model of DSRCT, harbor the t(11;22)(p13;q12) translocation, which underlies this tumor and genetic events triggering malignant transformation. Next, we investigated all the EWS-WT1 isoforms expressed in the DSRCT cell line model, and found six different transcripts. Thus, JN-DSRCT-1 cells display a broadly heterogeneous transcript population. Then, having demonstrated that JN-DSRCT-1 cells are particularly sensitive to trabectedin, with a strong G2/M block, we investigated whether the drug affected the binding of EWS-WT1 chimera to DNA sequence within target promoters, like the FUS-CHOP chimera in MLS disease or the EWS-FLI1 in Ewing Sarcoma [8, 20]. We found that EWS-WT1 transcript levels are affected by trabectedin treatment, to a slight but significative degree, and that the drug causes a quantitative decrease in the binding of the aberrant trascription factor to its target promoters. Therefore the decrease of chimera binding might be due at least in part to the decrease in EWS-WT1 transcripts produced in the cell after drug treatment, not only to a direct effect of trabectedin on the binding. However, expression profiling results suggested that trabectedin has significant effects on the transcriptional programs of JN-DSRCT-1 cells: in particular genes involved in apoptosis are induced and genes involved in proliferation are inhibited at early time points after drug treatment.

## Conclusions

JN-DSRCT-1 cells expressing the chimeric fusion products are sensitive to trabectedin; they show significant reduction of mRNA levels of the chimeric protein and decreased binding of EWS-WT1 chimera on its target promoters. The heterogeneity of EWS-WT1 transcripts represents an obstacle to the precise definition of the mode of action of trabectedin or other potential drugs, indicating the need of other cellular models for this disease. In this respect, recently other cell lines derived from DSRCT have been reported [21]; these cells could be used to verify the activity and the mechanism of action of trabectedin.

## Additional files

Additional file 1: Sanger sequencing primers. Schematic representation of the primers used for PCR and Sanger sequencing. (PPTX 302 kb)
Additional file 2: RT-Q-PCR primers. Table of RT-Q-PCR primers used. (XLSX 10 kb)
Additional file 3: Trabectedin clustering analysis. Unsupervised clustering analysis of trabectedin treated samples and control samples. The colors of the tree branches indicate distinct groups calculated by the clustering algorithm. Green boxes around the sample names indicate control samples, while red boxes show trabectedin-treated samples. (PPTX 68 kb)
Additional file 4: DEGs after trabectedin treatment of JN-DSRCT-1 cells. Differentially expressed genes for the three time points of JN-DSCRT-1 treatment with trabectedin (6 h, 12 h, 24 h). logFC, log2-fold change; AveExpr, average expression across the samples; t and B, results from the statistical test; *P*. Value, raw *p*-value from the statistical test; adj.P.Val, FDR adjusted *p*-values. (XLSX 318 kb)
Additional file 5: Pathway analysis after trabectedin treatment of JN-DSRCT-1 cells. Pathway analysis for the differentially expressed genes 6 h after trabectedin wash-out. pSize, number of genes belonging to the pathway; tA, statistical measurement of pathway activation (if greater than 0) or inhibition (if lower than 0), pGFdr, FDR adjusted *p*-value. (XLSX 39 kb)

## Abbreviations

ChIP: Chromatin immuno precipitation
DEGs: Differentially expressed genes
DSRCT: Desmoplastic small round cell tumor
FISH: Fluorescence in situ hybridization
GEP: Gene expression profiling
