# Supplementary figures and images for: Mechanism of action of trabectedin in desmoplastic small round cell tumor cells
Source: BMC Cancer. 2017 Feb 6;17:107. doi: 10.1186/s12885-017-3091-1 (PMC5294815; doi:10.1186/s12885-017-3091-1)

## Slide 1
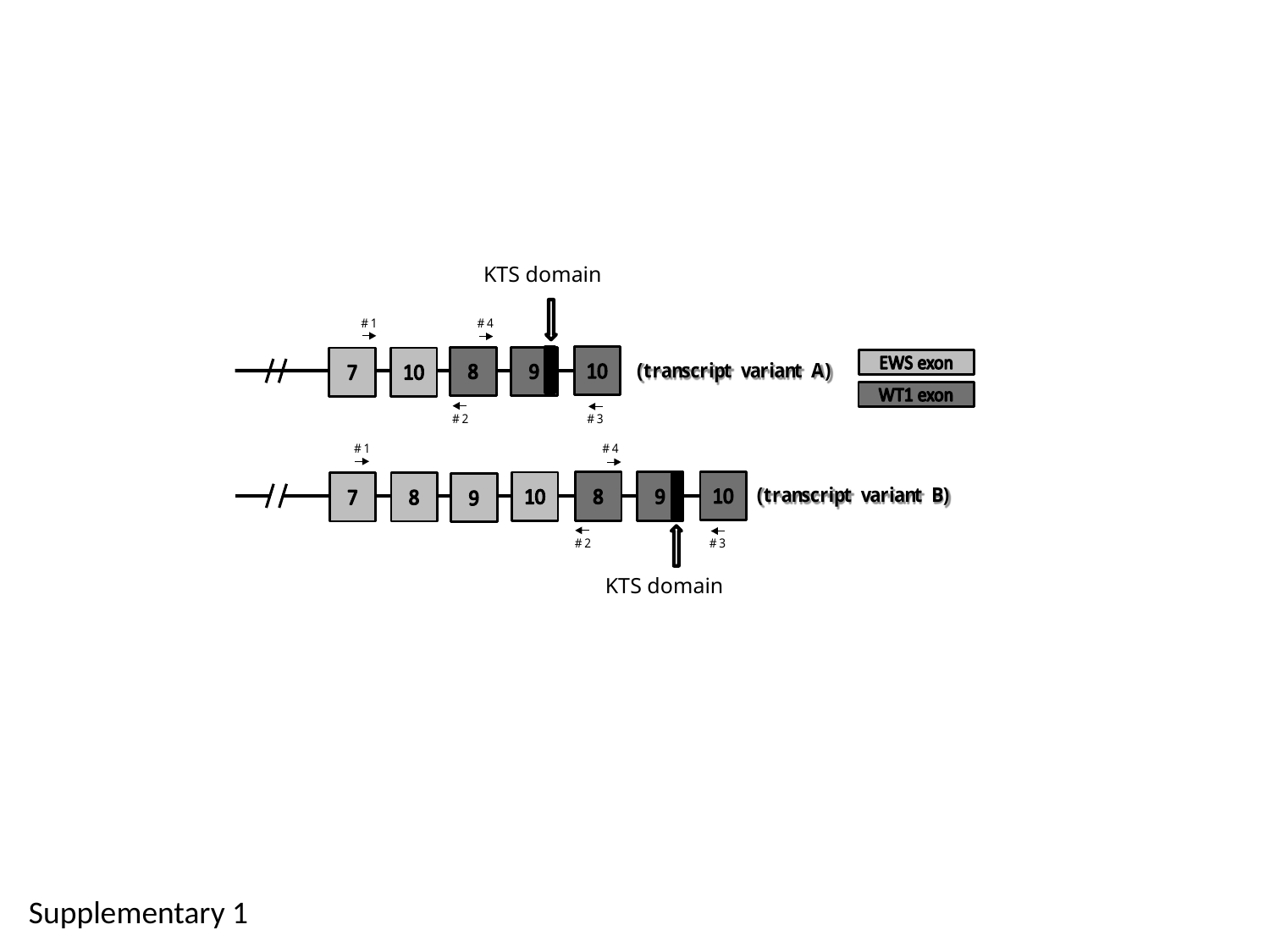

KTS domain
KTS domain
Supplementary 1

Supplement: Additional file 1: — Sanger sequencing primers. Schematic representation of the primers used for PCR and Sanger sequencing. (PPTX 302 kb) [file 12885_2017_3091_MOESM1_ESM.pptx]

## Slide 1
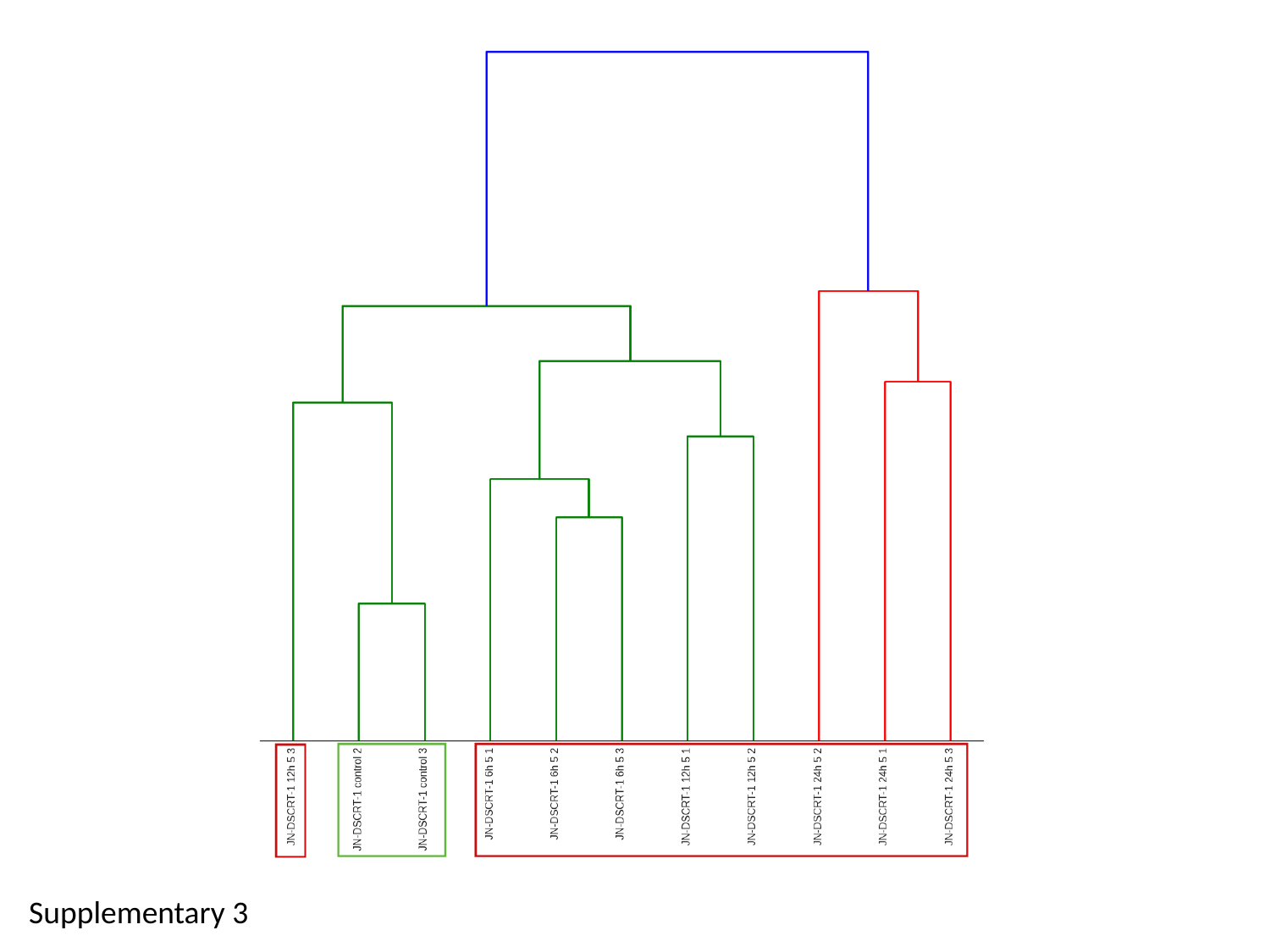

Supplementary 3

Supplement: Additional file 3: — Trabectedin clustering analysis. Unsupervised clustering analysis of trabectedin treated samples and control samples. The colors of the tree branches indicate distinct groups calculated by the clustering algorithm. Green boxes around the sample names indicate control samples, while red boxes show trabectedin-treated samples. (PPTX 68 kb) [file 12885_2017_3091_MOESM3_ESM.pptx]
